# Host sympatry and body size influence parasite straggling rate in a highly connected multihost, multiparasite system

**DOI:** 10.1002/ece3.2971

**Published:** 2017-04-17

**Authors:** Jose L. Rivera‐Parra, Iris I. Levin, Kevin P. Johnson, Patricia G. Parker

**Affiliations:** ^1^ Department of Biology and Whitney R. Harris World Ecology CenterUniversity of Missouri—St LouisSt LouisMOUSA; ^2^ Departamento de PetróleosFacultad de Geología y PetróleosEscuela Politécnica NacionalQuitoEcuador; ^3^ Illinois Natural History SurveyUniversity of IllinoisChampaignILUSA; ^4^ Saint Louis Zoo WildCare InstituteOne Government DriveSaint LouisMOUSA; ^5^ Present address: Department of BiologyAgnes Scott CollegeDecaturGAUSA

**Keywords:** Galapagos, host breadth, host switching, lice, parasite speciation, seabirds

## Abstract

Parasite lineages commonly diverge when host lineages diverge. However, when large clades of hosts and parasites are analyzed, some cases suggest host switching as another major diversification mechanism. The first step in host switching is the appearance of a parasite on an atypical host, or “straggling.” We analyze the conditions associated with straggling events. We use five species of colonially nesting seabirds from the Galapagos Archipelago and two genera of highly specific ectoparasitic lice to examine host switching. We use both genetic and morphological identification of lice, together with measurements of spatial distribution of hosts in mixed breeding colonies, to test: (1) effects of local host community composition on straggling parasite identity; (2) effects of relative host density within a mixed colony on straggling frequency and parasite species identity; and (3) how straggling rates are influenced by the specifics of louse attachment. Finally, we determine whether there is evidence of breeding in cases where straggling adult lice were found, which may indicate a shift from straggling to the initial stages of host switching. We analyzed more than 5,000 parasite individuals and found that only ~1% of lice could be considered stragglers, with ~5% of 436 host individuals having straggling parasites. We found that the presence of the typical host and recipient host in the same locality influenced straggling. Additionally, parasites most likely to be found on alternate hosts are those that are smaller than the typical parasite of that host, implying that the ability of lice to attach to the host might limit host switching. Given that lice generally follow Harrison's rule, with larger parasites on larger hosts, parasites infecting the larger host species are less likely to successfully colonize smaller host species. Moreover, our study supports the general perception that successful colonization of a novel host is extremely rare, as we found only one nymph of a straggling species, which may indicate successful reproduction.

## 1 Introduction

Colonization of novel environments can lead to the effective interruption of gene flow and generation of novel species (Feder, Egan, & Forbes, 2012; Ogden & Thorpe, 2002; Schluter, 2009). Fragmented and isolated habitats, such as oceanic archipelagos like the Galapagos or Hawaiian Islands, have been important in our understanding of the mechanisms of adaptive radiation and speciation by genetic drift (e.g., Grant & Grant, 2002). Parasite populations are fragmented naturally by having the host body as habitat. Thus, understanding what conditions limit the host breadth of parasites and under which circumstances they can overcome those barriers is key to understanding parasite diversification. Furthermore, this information is fundamental to understanding the potential for parasite adaptation to local host community changes and risk of co‐extinction with their host.

Evidence suggests that a major mechanism for parasite speciation is cospeciation (Cooper, Griffin, Franz, Omotayo, & Nunn, 2012; Demastes et al., 2012; Hughes, Kennedy, Johnson, Palma, & Page, 2007; Huyse, Poulin, & Théron, 2005; Koop, DeMatteo, Parker, & Whiteman, 2014), which occurs when a parasite lineage speciates simultaneously with its host (Huyse et al., 2005; Koop et al., 2014). Another major mechanism underlying parasite diversification is host switching (Clayton & Johnson, 2003; Johnson, Williams, Drown, Adams, & Clayton, 2002), in which a subset of a parasite population successfully colonizes a new host species and then subsequently becomes isolated from populations on the original host. Previous studies of avian louse cophylogenetics in different systems have found evidence for both cospeciation (Hughes et al., 2007) and ancient host switching (Johnson, Weckstein, Witt, Faucett, & Moyle, 2002) that may explain current patterns of parasite diversity. A challenge for identifying host switching in cophylogenetic analyses is pinpointing the conditions under which the host switching began. Host switching is suggested to start by expansion of host breadth where straggling individuals establish a breeding population on a novel host and later colonize other individuals in the novel host population (Norton & Carpenter, 1998; Paterson & Gray, 1997; Ricklefs, Fallon, & Bermingham, 2004). Straggling parasites are individuals that ended up on the “wrong host” but, commonly, do not survive or establish breeding populations on that host (Rozsa, 1993). Whiteman, Santiago‐Alarcon, Johnson, and Parker (2004) provided insight into some of the factors behind straggling parasites from goats (*Capra hircus*) and Galapagos doves (*Zenaida galapagoensis*) on Galapagos hawks (*Buteo galapagoensis*). They suggested that the scavenging behavior of hawks on goat carcasses and predation on doves provided the opportunities for parasites to end up on this atypical host. In this study, we performed an analysis of the conditions involved in parasite straggling events in a highly spatially connected and phylogenetically closely related multihost, multiparasite system and looked for evidence of cases where breeding populations of parasites were established on atypical hosts and analyzed the factors behind specificity.

Our study focuses on ectoparasitic lice infecting five species of seabirds in the Galapagos Islands. We studied the ischnoceran *Pectinopygu*s spp. feather lice, as well as the amblyceran *Colpocephalum* spp. body lice. These two groups of lice are obligate ectoparasites that complete their life cycles on their hosts. Ischnoceran lice feed on feathers are considered poor dispersers and are characterized as highly host specific (Price, Hellenthal, Palma, Johnson, & Clayton, 2003). The main defense that birds use to deal with these parasites is preening (Bush & Clayton, 2006; Bush, Sohn, & Clayton, 2006; Johnson, Bush, & Clayton, 2005). Because they are more mobile off the host, amblyceran lice are considered better dispersers and less host specific than ischnoceran lice (Clayton, Gregory, & Price, 1992). Amblyceran lice feed on skin tissue and may rupture the skin to feed on blood, where they might interact with the immune system of the host (Johnson, Weckstein, Bush, & Clayton, 2011; Johnson et al., 2005; Whiteman, Matson, Bollmer, & Parker, 2006). In both cases (amblycera and ischnocera), the way these parasites escape host preening is by firmly attaching to different components of the host feathers. For example, avian wing lice escape host preening by inserting their bodies between the feather barbs of the wing feathers. Johnson et al. (2005) and Bush et al. (2006) found that, in the case of ischnoceran lice, the match between the space between wing feather barbs and louse body width was critical for their ability to effectively escape host preening defenses and survive on the host. In the case of amblyceran lice that live closer to the skin, they attach to fibers of the downy undercover feathers using their mandibles, but the specific relationship between feather components and louse attachment is not as clear as for ischnoceran lice (Johnson et al., 2005).

The research presented here is relevant to understanding how host switching begins and what factors are behind the speciation and diversity of parasites, particularly ectoparasitic lice. Our driving hypotheses were as follows: (1) The colonial behavior of the hosts may have an effect on frequency and directionality of host switching; and (2) the ecomorphology of louse attachment may be another key factor in opportunities for host switching. We predicted that (1) host switching frequency would be higher in populations nesting in dense multispecies colonies; and (2) parasites smaller than the lice species commonly found on the host would have a higher frequency of host switching than parasites larger than the typical lice species. The specific objectives of this study were to (1) describe the occurrence of straggling events across mixed seabird breeding colonies; (2) analyze the effect of the local host species composition on the frequency of straggling events; (3) test the effects of relative host density within a mixed seabird colony on the prevalence of straggling lice; (4) analyze directionality in straggling events, related to louse attachment efficiency; and (5) test for evidence of louse breeding on the new host in cases where adult straggling lice were found.

## 2 Materials and Methods

### 2.1 Seabirds from the Galapagos Islands and their ectoparasitic lice

**Figure 1:**
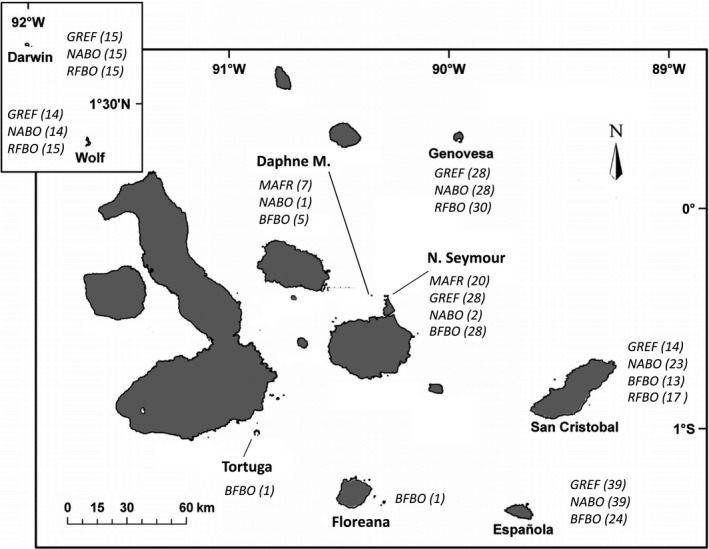
Map of the study area indicating the local host community composition and the number of hosts sampled on each island. Great frigatebird (GREF), magnificent frigatebird (MAFR), Nazca booby (NABO), red‐footed booby (RFBO), and blue‐footed booby (BFBO)

Our study took place on the Galapagos Islands, located in the Pacific Ocean off the West coast of Ecuador. We sampled seven islands across the archipelago, which represent the major breeding colonies of the five host species included in the study. Specifically, we sampled the northern islands of Darwin, Wolf, and Genovesa, the central islands of North Seymour and Daphne Major, and the eastern islands of Española and San Cristobal. Figure 1 summarizes the sampled islands, local host community composition and hosts sampled from each island. Our study system included three species of boobies: blue‐footed (*Sula nebouxii*), Nazca (*S. granti*) and red‐footed (*S. sula*), and two frigatebirds: great (*Fregata minor*) and magnificent (*F. magnificens*). All of these species are colonial breeders, but they differ in key aspects of their natural history. Frigatebirds are kleptoparasites of other birds, and they harass other individuals to steal their catch, or catch fish by skimming the surface of the water, whereas boobies catch fish by plunge diving. Both frigatebird species and red‐footed boobies nest in trees, bushes, or shrubs, whereas Nazca and blue‐footed boobies nest on the ground, with blue‐footed boobies preferring nesting sites farther inland and in more sandy areas, compared to the rocky areas near cliffs favored by Nazca boobies (Del Hoyo, Elliott, & Sargatal, 1992). Even when they are not territorial, each breeding pair will defend the area close to its nest (Del Hoyo et al., 1992), which causes them to physically interact with passing or landing neighbors, probably creating chances to exchange parasites.

On these host species, we identified a total of seven ectoparasitic lice (Phthiraptera) species from two different suborders: ischnocera and amblycera. Table 1 summarizes typical host–parasite associations and overall sample numbers for each parasite and each host (based on Price et al., 2003; Rivera‐Parra, Levin, & Parker, 2014). For the purposes of this study, we define a “typical” host as the one implicated in the host–parasite association commonly reported in the literature; for example, the typical host of *Pectinopygus annulatus* is the Nazca booby (Table 1).

**Table 1:** Summary of typical host–parasite associations. Parentheses indicate the overall sample size of each host and parasite species

Host	Ischnocera	Amblycera
Great frigatebird (*Fregata minor*) – (138)	*Pectinopygus gracilicornis* (1,505)	*Colpocephalum angulaticeps* (914)
Magnificent frigatebird (*F. magnificens*) ‐ (27)	*P. fregatiphagus* (405)	*C. spineum* (56)
Nazca booby (*Sula granti*) – (122)	*P. annulatus* (1,195)	
Blue‐footed booby (*S. nebouxii*) – (72)	*P. minor* (763)	
Red‐footed booby (*S. sula*) – (77)	*P. sulae* (1,055)	

Rivera‐Parra et al. (2014), working in this same system, found that all parasite species included in this study had a prevalence higher than 85%. Furthermore, when analyzing the intensity of infection, they found that ischnoceran *Pectinopygus* sp. lice showed higher intensities than the amblyceran *Colpocephalum* sp. Among the *Pectinopygus* sp. lice, the highest intensity of infection was found on *Pectinopygus fregatiphagus*, which infects magnificent frigatebirds, with a median of 24 lice per host, whereas the other *Pectinopygus* sp. showed a median intensity of infection between 7 and 10 lice per host.

We sampled five host species from seven islands in the Galapagos Archipelago (Figure 1). We captured the birds by hand and performed a modified dust‐ruffling protocol to collect the ectoparasites (details on sampling methods and precautions taken to avoid cross‐contamination can be found at Rivera‐Parra et al., 2014). We used a pyrethrin‐based flea powder (Zodiac, pyrethrin 1%, Wellmark International, Schaumburg, Illinois) and ruffled the bird a maximum of three times. We applied a standard amount of flea powder (~6 g) and waited a standard time (1 min) between ruffling bouts. We recorded the species of each bird and sex, and later we confirmed this putative identification using molecular techniques (detailed below). When we sampled a bird that was nesting, we recorded the number of nests within ten meters of the focal nest, distance to the nearest nest, and the species identity at each nest within ten meters.

We stored the collected ectoparasites in leak‐proof tubes with 95% ethanol for later identification. We used the identification key found in Price et al. (2003) and specimens identified by R. Palma as reference to sort the collected lice to species level. In cases where there were no conspicuous morphological differences, for example, *Pectinopygus gracilicornis* and *P. fregatiphagus*, we used a molecular identification approach to confirm the species identification.

We extracted DNA following the voucher method (Cuickshank et al., 2001), using a Macherey‐Nagel tissue extraction kit (Macherey‐Nagel, Duren, Germany). We incubated each individual louse, which had previously been cut between the head and the thorax, in proteinase K for 72 hr at 55°C and then followed the extraction protocol from the kit, with two sequential elutions, each with 20 μl of warm buffer at 70°. We sequenced a 300‐bp fragment of the mitochondrial gene *cytochrome oxidase subunit I* (COI), using the primers L6625 (5′‐COG GAT CCT TYT GRT TYT TYG GNC AYC C‐3′) and H7005 (5′ –CCG GAT CCA CAN CRT ART ANG TRT CRT G‐3′; Hafner et al., 1994). The specific PCR reagent conditions were 1× MgCl_2_, 1.5 mmol/L of MgCl_2_, 0.2 mmol/L of each dNTP, 0.08 mg/ml of BSA, 0.625 units of DNA polymerase, and 1 μl of stock DNA. The specific amplification conditions were initial denaturation at 94°C for 2 min, then 35 cycles of: 94°C for 30 s, 46°C for 30 s, and 72°C for 30 s, and then a final extension at 72°C for 7 min. PCR products were visualized in a 1.5% agarose gel and then cleaned using ExoSap (USB Scientific, Cleveland, OH, USA). We sequenced both chains of the products using BigDye terminator kit v3.1 (Applied Biosystems, Foster City, CA, USA). Sequencing products were run in an automatic sequencer ABI 3130xI and contigs were assembled using SeqManII v.4 (DNAStar, Madison, WI, USA). Sequences were aligned using Clustal W, part of Mega V5.05 (Tamura et al., 2011). In the case of the *Pectinopygus* spp. parasites, we used reference sequences from Hughes et al. (2007; GenBank accession numbers: *Pectinopygus gracilicornis* DQ482969, *P. fregatiphagus* DQ489433, *P. annulatus* DQ482970; *P. minor* DQ482966; *P. sulae* DQ482971) for each parasite species. We followed Rivera‐Parra et al. (2014) for the identification of the *Colpocephalum* spp. parasites. We tested for the best fitting evolutionary model using MEGA V5.05 (T92 +  G for *Pectinopygus* spp. parasites and T92 for *Colpocephalum* spp. lice) and then constructed maximum‐likelihood trees with 1,000 bootstrap pseudoreplicates using MEGA V5.05 (Tamura et al., 2011). To test for the presence of nymphs corresponding to the same species of straggling adults, we followed the same phylogenetic method described above and confirmed the species identity of each individual nymph based on phylogenetic analysis.

We calculated prevalence and distribution of straggling events based on host species, parasite species, and island. After using both morphology and molecular techniques to confirm parasite species identity, we performed chi‐square tests in SPSS v13.0 (SPSS Inc., Chicago, IL, USA) to test for the effect of island community composition, relative host density within a mixed breeding colony, and louse body size, on the frequency of straggling events. We conducted Spearman's rho correlations with 1,000 bootstrap repetitions to test for the association between the presence of straggling lice with distance to the nearest nest, number of conspecific nests within 10 m of the focal nest, and number of heterospecific nests within ten meters of the focal nest.

## 3 Results

We sampled a total of 436 host individuals. Of those, 26 (5.65%) had straggling adult lice; 14 had only straggling ischnocera, nine had only straggling amblycera, and three had both types of straggling parasites. From the parasite perspective, we analyzed 3,564 *Pectinopygus* spp. lice and found 23 straggling individuals (0.65%). In the case of the *Colpocephalum* spp. parasites, of 970 analyzed lice, 15 straggling lice were found (1.55%). There is a significant difference in the frequency of straggling individuals between Amblyceran and Ischnoceran lice (*t*‐test = 2.72; *p* < .05). Table 2 summarizes the frequency of straggling *Pectinopygus* parasites and the species identity of the straggling lice per each host species per each island, together with the identity of the straggling parasites found on each host species. In the case of *Pectynopigus* spp. lice, the median number of straggling lice found on each host was 1 (hosts = 17; mean = 1.35), and no more than three straggling lice were found on a single host sample. The specific numbers of straggling *Colpocephalum* spp. lice found on each host from each island and the specific species identity of such lice can be reviewed in Table 3. For the *Colpocephalum* lice, the median of straggling lice per host was 1 (hosts = 11; mean = 1.36) and the maximum straggling lice found on a single host was 3.

**Table 2:** Summary of straggling ischnoceran lice., showing the number of hosts with straggling lice on them on each island and, in parentheses, the number of *Pectinopygus* parasites found on each host on each island and its species identity. PFREG = *P. fregatiphagus*, PGRA = *P. gracilicornis*, PMIN = *P. minor* and PSUL = *P. sulae*

	*Sula granti*	*Sula nebouxii*	*Fregata magnificens*	Total
Darwin	3 (2 PSUL, 1 PGRA)			3 (2 PSUL, 1 PGRA)
Wolf	3 (5 PSUL)			3 (5 PSUL)
Genovesa	4 (4 PSUL, 1 PGRA)			4 (4 PSUL, 1 PGRA)
Daphne M.	1 (2 PSUL)		3 (1 PSUL, 1 PMIN, 1 PGRA)	4 (3 PSUL, 1 PMIN, 1 PGRA)
N. Seymour	1 (3 PGRA)	2 (2 PFRE)		3 (3 PGRA, 2 PFRE)
TOTAL	12 (13 PSUL, 5 PGRA)	2 (2 PFRE)	3 (1 PSUL, 1 PMIN, 1 PGRA)	17 (14 PSUL, 6 PGRA, 2 PFRE, 1 PMIN)

**Table 3:** Summary of straggling amblyceran lice., showing the number of hosts with straggling lice on them on each island and, in parentheses, the number of *Colpocephaulm* parasites found on each host on each island and its species identity. CANG = *C. angulaticeps*, CSPI = *C. spineum*

	*Sula granti*	*Sula nebouxii*	*Sula sula*	*Fregata magnificens*	Total
Wolf			1		1
			2 CANG		2 CANG
Genovesa			1		1
			1 CANG		1 CANG
Española		1			1
		1 CANG			1 CANG
S. Cristobal		1	3		4
		1 CANG	3 CANG		4 CANG
Daphne M.				1	1
				2 CANG	2 CANG
N. Seymour	1	2			3
	3 CANG	1 CANG, 1 CSPI		4 CANG, 1 CSPI	
TOTAL	1	4	5	1	11
	3 CANG	3 CANG, 1 CSPI	6 CANG	2 CANG	14 CANG, 1 CSPI

On average, the closest nest was at 11.46 m for blue‐footed boobies, 4.37 m for great frigatebirds, 3.78 m for Nazca boobies, 3.71 m for red‐footed boobies, and 2.27 m for magnificent frigatebirds. The average number of nests of conspecifics within 10 m was 8.63 for Nazca boobies, 5.28 for great frigatebirds, 3.20 for red‐footed boobies, 2.48 for magnificent frigatebirds, and 1.09 for blue‐footed boobies. The average number of nests of heterospecifics (any other host species sampled in this study) within 10 m of the focal nest was 1.58 for red‐footed boobies, 1.43 for great frigatebirds, 1.09 for magnificent frigatebirds, 0.53 for blue‐footed boobies, and 0.33 for Nazca boobies. Therefore, Nazca boobies were found in dense colonies and were predominantly surrounded by conspecifics, while red‐footed boobies and both species of frigatebirds were more likely to be found in colonies overlapping with those of other species. The islands that showed the highest degree of spatial overlap were Darwin, where red‐footed boobies and great frigatebirds nest in overlapping areas; and Wolf, where Nazca and red‐footed boobies were nesting in intersecting areas. A caveat is that these measurements consider only the breeding population (nests), are just a snapshot of the whole breeding season, and do not include resting or roosting birds that were not breeding at the time of sampling.

The local host community composition explained the frequency of parasite straggling events. First, we analyzed all the straggling lice and found that 19 of 23 ischnoceran straggling events happened on islands where the typical host was present (χ^2^ = 9.78, *df* = 1, *p *=* *.002 ± 0.001 95%CI). In the case of amblyceran lice, 13 of 15 straggling events happened on islands where the typical host was present (χ^2^ = 8.07, *df* = 1, *p *=* *.006 ± 0.002 95%CI). When both types of lice were combined, we found that 32 of 38 events were found on islands where the typical host was present (χ^2^ = 17.79, *df* = 1, *p *<* *.0001 ± 0 95%CI). We did not find relationships between the presence of straggling lice and distance to the nearest nest (*p *=* *.95), number of conspecific nests within 10 m (*p *=* *.106), or number of heterospecific nests within 10 m (*p *=* *.676).

We counted seven host individuals that had straggling lice and were breeding at the time of sampling. We tested whether the specific spatial location within a mixed breeding colony had an effect on the species identity of the straggling lice. Specifically, we asked whether the species identity of the straggling lice was explained by the presence of the typical host within 10 m of the host where a straggling louse was found. We found that the presence of the typical host within 10 m of the sampled host did not have a significant effect on explaining the presence of straggling ischnoceran lice (χ^2^ = 1.8, *df* = 1, *p *=* *.377 ± 0.012 95%CI), amblyceran lice (χ^2^ = 1.8, *df* = 1, *p *=* *.375 ± 0.012 95%CI), or for any straggling event (both parasites combined: χ^2^ = 4.5, *df* = 1, *p *=* *.64 ± 0.06 95%CI).

We analyzed potential directionality in the straggling events. We asked whether the ability to escape from host preening defenses related to a match between louse body width and host size (Bush et al., 2006; Johnson et al., 2005), and whether this could explain the frequency of different straggling events in ischnoceran lice. We predicted that if louse escape ability had a significant effect on straggling frequency, then only parasites smaller than the typical parasite of each host would be found as stragglers, because smaller lice could potentially insert between larger feather barbs, but not the other way around. When the ischnoceran parasite species are ranked based on their head width, thorax width, and abdomen width, they rank as follows, largest to smallest: *Pectinopygus annulatus* (Nazca booby), *P. minor* (blue‐footed booby), *P. sulae* (red‐footed booby) and the parasites that infect frigatebirds *P. fregatiphagus* (magnificent frigatebird) and *P. gracilicornis* (great frigatebird). We found significant differences in the direction of straggling events, which supported this hypothesis. Of 23 straggling lice, 20 were found on a host that usually harbors larger‐bodied parasites (χ^2^ = 12.56, *df* = 1, *p* = 0 ± 0 95% CI).

We found 12 individual birds that had nymphs as well as straggling adult ischnoceran lice. We tested a total of 58 nymphs and found one case of one nymph from the straggling louse species on the novel host. Specifically, we found adults and a nymph of *Pectinopygus gracilicornis* (which is normally found on great frigatebirds) on a Nazca booby from Genovesa.

## 4 Discussion

We have documented straggling events throughout the seabird and louse community of the Galapagos Archipelago. We also found evidence of the presence of adults considered as stragglers on a novel host and, in one case, a nymph of a straggling species on the atypical host. This might indicate the early steps in successful host breadth expansion. Furthermore, the likelihood of survival on a novel host might be directly driven by specific eco‐morphological adaptation to escape from host preening defenses in ischnoceran lice.

Straggling events may happen during any physical contact between host species, for example, landing and bumping into other hosts, roosting together, or kleptoparasitism by frigatebirds. Furthermore, the typical (original) host of the straggler was present on the island for a significant proportion of straggling cases, supporting that the “jump” to an atypical host often happens within a local vicinity. Most of the straggling ischnoceran lice corresponded to *Pectinopygus fregatiphagus* or *P. gracilicornis* (Table 2), which infect great and magnificent frigatebirds, respectively, and most of these lice were found on red‐footed boobies. Moreover, most of the *Colpocephalum* amblyceran lice that commonly infect frigatebirds were found on red‐footed boobies as well (Table 3). Frigatebirds are kleptoparasites that harass other birds to steal their catch (Del Hoyo et al., 1992). Observations during our field work suggest that among the three booby species considered in this study, the most heavily parasitized by frigatebirds are red‐footed boobies, which are the smallest of the three booby species (see also Le Corre & Jouventin, 1997). Specifically, frigatebirds harass other birds by pecking and plucking feathers from above while both are in flight (Osorno, Torres, & Macias Garcia, 1992); body contact can occur during these events and it is likely that parasites can move to the bird being parasitized by the frigatebirds. We found significantly more amblyceran lice as stragglers than ischnoceran lice, which might be explained by differences in their natural history. The ischnoceran lice that often insert between feathers barbs are much less mobile off the host than amblyceran lice, which are more mobile and found more often in downy feathers. Thus, it is likely that during strong physical interactions, amblyceran lice are more easily dislodged than ischnoceran lice that are embedded within host feathers (see also Johnson et al., 2011).

There were few cases in which the typical host of the straggling lice was not found on the same island where the host was sampled. Specifically, we found one Nazca booby sampled on Daphne Major that had *Pectinopygus sulae*, which is typically found on red‐footed boobies, and two cases of magnificent frigatebirds, one that had *P. sulae* and other that had *P. gracilicornis* (which typically infects great frigatebirds). Both typical hosts, red‐footed booby and great frigatebird, were not found on Daphne Major during our fieldwork, nor have they been reported as present on the island (Swash & Still, 2005; C. Valle, personal communication). Daphne Major is a small island in the middle of the archipelago, separated by ~10 km from North Seymour, where there is another large colony of magnificent frigatebirds sympatric with a colony of great frigatebirds (Anderson, 1989; C. Valle, personal communication, observations from this study). There are no studies on the connection between these colonies, but it is likely that such vagile birds as magnificent frigatebirds may move between these nearby islands. Thus, the great frigatebird lice found on a magnificent frigatebird on Daphne Major may have come originally from a great frigatebird from North Seymour. More intriguing are the cases where we found *P. sulae*, which typically infects red‐footed boobies, on a Nazca booby individual from Daphne Major. Nazca boobies and red‐footed boobies overlap on several islands (Darwin, Wolf, Genovesa and San Cristobal). Genetic evidence suggests that red‐footed boobies and Nazca boobies move significantly between some island pairs within the archipelago (Levin & Parker, 2012; P. Baiao and P. G. Parker, unpublished data). Thus, the straggling lice may have been acquired during these movements. Also, birds could move for reasons not associated with breeding; juveniles might be prospecting, for example.

Our results suggest the eco‐morphology of lice escape behavior is an important factor behind straggling events. Bush et al. (2006) and Johnson et al. (2005) documented how lice larger than the space between barbules (feather components) had lower survivorship than parasites the same width or smaller than this space. We found that straggling events happen significantly more often if the straggling louse is smaller than the typical parasite commonly found on a given host. There is the possibility that parasites larger than the typical parasite have a similar rate of straggling but they do not survive long enough to be detected. Parasites on the upper extreme of Harrison's rule, meaning the largest parasites found on the largest host of the community, may be at an evolutionary dead end, where they cannot effectively survive on or successfully colonize any other host of the community. Thus, such parasite species are at greater risk of co‐extinction with their hosts (Koh et al., 2004).

The relationship of feather components and the way amblyceran lice attach to their hosts and avoid death during preening is less well understood than for the ischnoceran lice (Johnson et al., 2005). The accepted mechanism is that amblyceran lice burrow and entangle themselves in the downy feathers closer to the host body or run away from host preening efforts. Frigatebirds, when compared to boobies, have overall fewer feathers and fewer inner downy feathers (J. L. Rivera‐Parra, personal observation), but they differ in their feeding behavior. It is likely that *Colpocephalum* that evolved on frigatebirds that do not plunge dive could not survive the dislodging forces during this feeding behavior, common to the three boobies. Therefore, it is likely that the *Colpocephalum* individuals found on boobies (particularly red‐footed boobies) might have been recently acquired during the approach to the island (and subsequent harassment by frigatebirds) and might die during the next fishing trip of the host. In this scenario, we may be underestimating straggling rates from frigatebird parasites that end up on boobies.

An important question is how to define a straggler versus a successful host‐switch or host breadth expansion (Rozsa, 1993; Whiteman et al., 2004). We considered the presence of nymphs as well as adults on an atypical host as the cutting point between straggling and successful host breadth expansion. We found evidence of nymphs of *P. gracilicornis* on a Nazca booby, together with adults of the same louse species, which suggests the presence of a breeding population of this parasite species on this host individual. This finding, together with an overall prevalence of straggling lice of ~1%, speaks of a prevalent phenomenon of parasites ending up “on the wrong host.” If speciation is driven by host switching, it would start with an isolate of the parasite species colonizing a novel host, expanding its host breadth and then diverging from the original species due to lack of gene flow (Clayton & Johnson, 2003; Rozsa, 1993). Moreover, for a successful host breadth expansion and later speciation, the transmission of this emerging parasite lineage is fundamental, as well as limited secondary contact with the original parasite population. Parasite populations are fragmented and have a relatively high risk of extinction (Nieberding & Olivieri, 2007); when the host dies, the whole parasite population resident on that host dies as well, unless it is a mobile parasite and/or a parasite with free‐living phases.

Transmission to other individuals in the case of parasites can be vertical (to offspring) or it is possible that it might be horizontal through social interactions such as during mating or territorial disputes (Clayton et al., 1992; Whiteman & Parker, 2004). This latter transmission might be limited by the presence of the typical parasite on the specific host (Bush & Malenke, 2008; Johnson, Malenke, & Clayton, 2009; Johnson et al., 2011). Thus, parasite‐free recently hatched chicks would be colonized by parasite species found on their parents. Then, depending on the population size, isolation of the population and stochastic events (e.g., death of hosts), something that started as a straggling event that established a breeding population on the novel host may lead to the displacement of the original typical parasite. By isolation from the source population, this process can lead to parasite speciation (Clayton & Johnson, 2003; Johnson, Williams, et al., 2002). This means such events are area specific, and therefore, it explains cases where parasite distribution differs across host range (Price et al., 2003). Moreover, this suggests that parasite diversity and specificity are maintained by stochastic events during transmission, where the most common parasite is the one that is transmitted to the next generation and across individuals.

In this study, we analyzed a system where we expected to find a significant number of straggling events, but we found few. Parasite specificity is very high and may respond to lice attachment, diving/feeding behavior of the host and small spatial separation even in dense seabird colonies. More research is needed to understand the exact mechanisms that maintain parasite specificity and diversity.

## Conflict of Interest

None declared.
